# Left Ventricular Thrombus After Atrial Myocardial Infarction in a Young Man With Preserved Global Ejection Fraction and Suspected Antiphospholipid Syndrome: A Case Report

**DOI:** 10.7759/cureus.113576

**Published:** 2026-07-29

**Authors:** Sohaib Minhas, Syed Waleem Pasha

**Affiliations:** 1 Cardiology, Wexham Park Hospital, Slough, GBR; 2 Cardiology, Lincolnshire Community Health Services NHS Trust, Lincoln, GBR

**Keywords:** anticoagulation, antiphospholipid syndrome, case report, lv thrombus, stroke

## Abstract

Left ventricular (LV) thrombus is known to occur following acute myocardial infarction (MI), generally seen when there is reduced left ventricular (LV) systolic function, and is an uncommon presentation in patients with preserved ejection fraction. We describe a case of a 28-year-old man with recurrent presentations with central chest pain in the weeks following primary percutaneous coronary intervention for an anterior ST-elevation MI (STEMI). Transthoracic echocardiography (TTE) revealed apical LV thrombi despite preserved systolic function. Coronary angiography confirmed patent left anterior descending artery stents without obstructive coronary artery disease. The patient's age, previous ischaemic stroke secondary to basilar artery thrombus, and absence of obvious significant cardiovascular risk factors raised suspicion for an underlying thrombophilic disorder. A thrombophilia screen was performed and was positive for lupus anticoagulant, suggesting antiphospholipid syndrome. Therapeutic anticoagulation was initiated with low-molecular-weight heparin bridging to warfarin. Follow-up TTE one month later demonstrated persistent LV thrombus, and continued imaging surveillance was arranged. He had follow up with haematology, who elected to keep him on long-term anticoagulation with warfarin. This case highlights that LV thrombus may develop despite preserved LV systolic function following MI and emphasises the importance of considering inherited or acquired thrombophilic conditions in young patients with recurrent thromboembolic events or unexpected LV thrombus. Early recognition of an underlying prothrombotic state may influence long-term anticoagulation strategies and reduce the risk of future embolic phenomena.

## Introduction

A 28-year-old gentleman who underwent primary percutaneous intervention (PPCI) for ST-elevation myocardial infarction (MI) one week ago presented with a one-day history of central chest pain. He was managed for Dressler's syndrome and discharged home. He presented three weeks later with cardiac-sounding chest pain and a new rise in troponin. On transthoracic echocardiogram, he was found to have an LV thrombus despite normal systolic function (ejection fraction (EF) of 55-60%) and was subsequently commenced on anticoagulation with warfarin.

LV thrombus typically occurs in individuals who have a reduced EF [1] as a result of stasis, endothelial injury, and a hypercoagulable state. Our patient was found to have no significant risk factors for ischaemic heart disease and had a normal systolic function. In addition to this, he had suffered an ischaemic stroke the year before. These facts raised suspicion of an underlying prothrombotic disease.

## Case presentation

A 28-year-old gentleman, with a background of left posterior inferior cerebellar artery ischaemic stroke secondary to left vertebral artery dissection with basilar artery thrombus, ventriculoperitoneal (VP) shunt, pericarditis, and recent STEMI, presented with central chest pain. This gentleman had a recent ST-elevation MI (STEMI) with percutaneous intervention (PCI) to the left anterior descending artery (LAD) the week prior to presentation. There was no history of atrial fibrillation or flutter. He was on treatment with aspirin 75 mg daily, clopidogrel 75 mg daily, ramipril 1.25 mg daily, and bisoprolol 3.75 mg daily. He was treated for Dressler's syndrome with colchicine 500 mcg twice daily and ibuprofen 400 mg three times a day and was scheduled for a transthoracic echocardiography (TTE).

**Figure 1:**
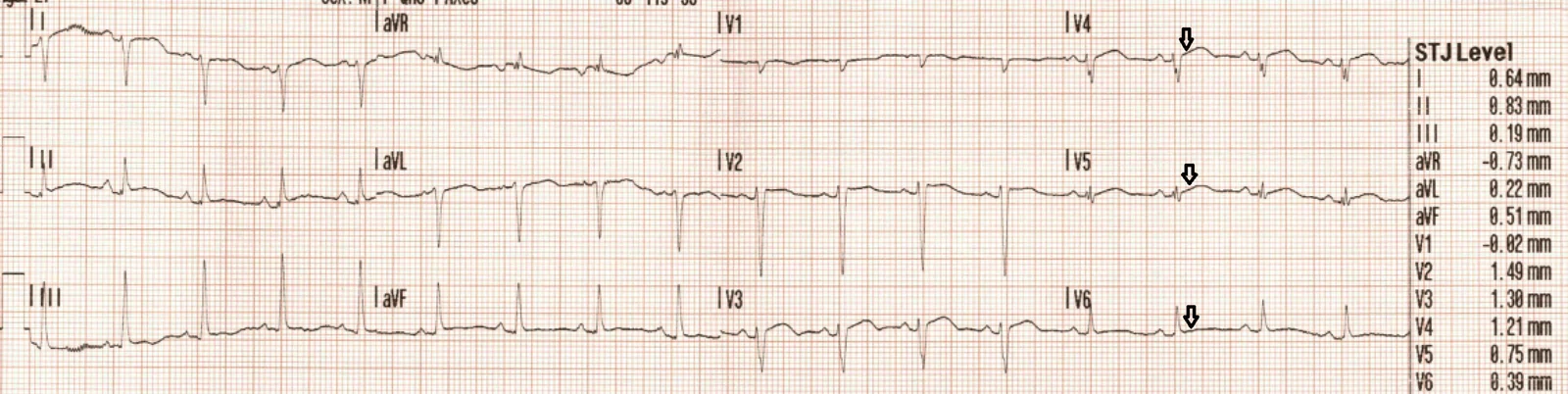
ECG - ST-elevation The arrows point to ST-elevation in leads V4-V6.

On review, his observations were normal, and the cardiovascular examination was unremarkable. Initial investigations revealed ST-elevation in leads V4-V6 on electrocardiogram (ECG) (Figure 1), which were unchanged in comparison to previous ECGs. High-sensitivity troponin I was elevated at 3,324 ng/L (0-33 ng/L) but had improved from an initial reading of 5,000 ng/L at the time of his recent STEMI presentation.

His investigations were as follows: haemoglobin of 166 g/L (130-180 g/L), platelets of 345 x 10^9^/L (150-450 x 10^9^/L), white cell count of 13.4 x 10^9^/L (4-11 x 10^9^/L), C-reactive protein of 3.4 mg/L (0-5 mg/L), estimated glomerular filtration rate >90 mL/min (90-120 mL/min). His total cholesterol was 3.2 mmol/L (<5 mmol/L), non-HDL cholesterol of 2.4 mmol/L (<4 mmol/L), triglycerides of 1.4 mmol/L (0-1.7 mmol/L), and LDL cholesterol of 1.7 mmol/L (<3 mmol/L).

Chest X-ray showed clear lung fields. He had a TTE demonstrating normal left ventricular size and function with a visually estimated ejection fraction of 55-60%, some suspicion of hypokinetic apical inferolateral wall, and normal wall thickness and filling pressures. Right ventricular size and function were normal, as well as the size of both atria. There was a trivial localised pericardial effusion and no evidence of thrombus.

**Figure 2:**
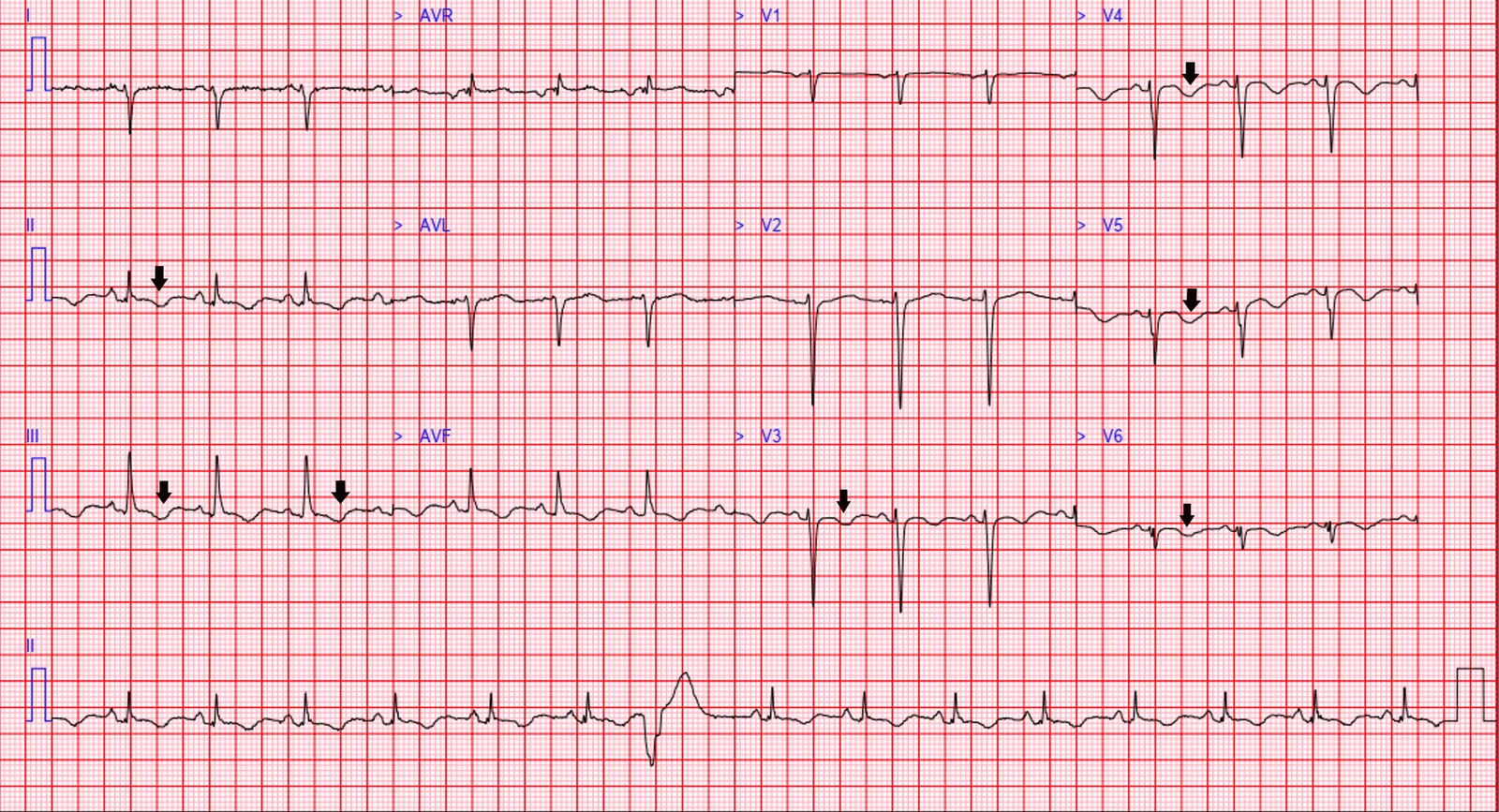
ECG - T-wave inversion The arrows point to T-wave inversion in leads II and III and V3-V6.

Three weeks later, this patient presented again with central chest pain and was found to have a dynamic rise in troponin from 135 ng/L to 376 ng/L (0-33 ng/L). ECG showed normal sinus rhythm with T-wave inversion in V3-V6, leads II and III, and aVF (Figure 2). He was scheduled for an angiogram and TTE.

**Figure 3:**
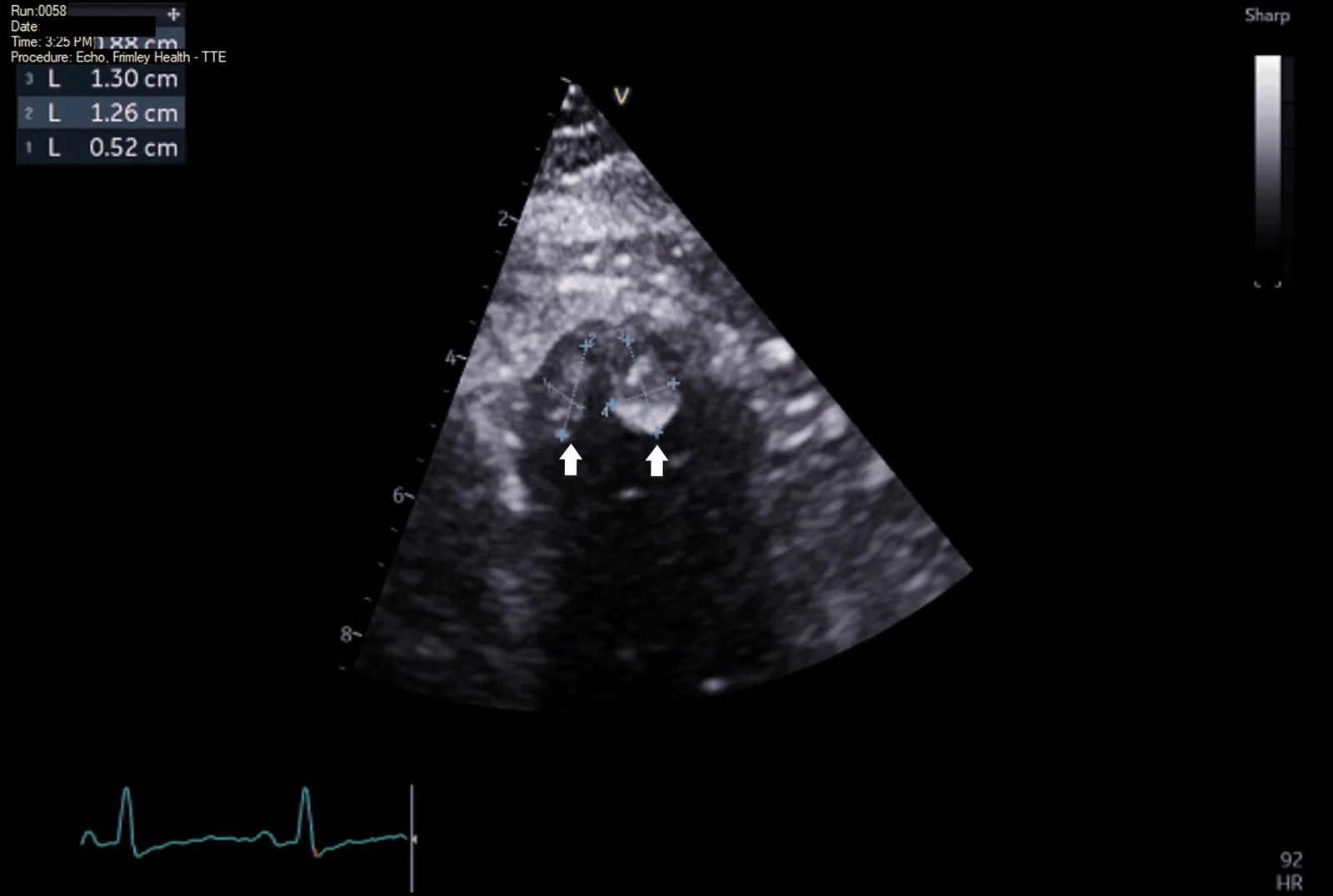
Echo - left ventricular (LV) thrombi The left arrow points to LV thrombus measuring 0.52 cm x 1.26 cm, and the right arrow points to LV thrombus measuring 1.30 cm x 0.8 cm.

An echocardiogram (Figure 3) revealed two apical LV thrombi (0.52 cm x 1.26 cm and 1.30 cm x 0.8 cm), with normal EF (55-60%) and an akinetic apical cup - all other parameters were unchanged from the previous echocardiogram. In the context of LV thrombus and a past history of a young patient with a history of basilar artery thrombus and ischaemic heart disease, underlying thrombophilia was considered. A thrombophilia screen was sent urgently, including anti-β2-glycoprotein I antibodies, protein C, protein S, factor V Leiden, lupus anticoagulant, and prothrombin gene mutation. Angiography revealed no obstructive coronary artery disease and patent LAD stents. The thrombophilia screen revealed a positive result for lupus anticoagulant on two tests conducted three months apart.

## Discussion

This brief review summarises key clinical contexts in which LV thrombus may occur, with emphasis on MI, reduced ejection fraction, thrombophilia, and oral contraceptive use. LV thrombus is a recognised complication following MI and is managed with anticoagulation and follow-up to reduce the risk of systemic embolism, including stroke [2].

Evidence from patients with acute MI supports this association. One study followed 105 unselected, consecutive patients after acute MI to assess the occurrence of LV thrombus; it found echocardiographic evidence of thrombus formation within the first two weeks in 40% of patients with anterior infarction and in none of the patients with inferior infarction [3].

Beyond infarct location, impaired ventricular function also appears to increase risk. In one study of 82 cardiac patients with an ejection fraction of 35% or less, 6.1% had LV thrombus on echocardiography, and 25.6% had spontaneous contrast, indicating increased stasis. Among the patients with LV thrombus, 80% had dilated cardiomyopathy secondary to ischaemic heart disease [1]. Similarly, a 2022 case report described a 63-year-old man with congestive heart failure of ischaemic aetiology who developed LV thrombus following decongestion and haemoconcentration, despite no recent MI [4].

Additional reports suggest that LV thrombus should also be considered in selected patients without reduced ejection fraction or ischaemic heart disease. A 2022 case report described a 36-year-old man with recurrent thrombosis, including ischaemic stroke and ischaemic limb, who was found to have LV thrombus on echocardiography despite normal ejection fraction and no history of ischaemic heart disease; his thrombophilia panel raised suspicion of antiphospholipid syndrome [5]. In young women taking oral contraceptive pills who present with systemic thromboembolic events or MI, investigation for LV thrombus may also be appropriate, as it is a rare but reported complication of oral contraceptive use [2].

## Conclusions

LV thrombus is strongly associated with reduced ejection fraction, particularly in the context of dilated cardiomyopathy secondary to ischaemic heart disease. Patients who develop LV thrombus without a history of ischaemic heart disease or cardiomyopathy should be investigated for other potential causes, including thrombophilic conditions, such as antiphospholipid syndrome or factor V Leiden. Other prothrombotic contributors, including medication-related causes, should also be considered. Early identification of prothrombotic conditions may reduce the risk of future thromboembolic events through appropriate treatment.
